# Synthesis of β-Ga_2_O_3_:Mg Thin Films by Electron Beam Evaporation and Postannealing

**DOI:** 10.3390/ma17194931

**Published:** 2024-10-09

**Authors:** Weitao Fan, Sairui Li, Wei Ren, Yanhan Yang, Yixuan Li, Guanghui Liu, Weili Wang

**Affiliations:** 1 School of Physical Science and Technology, Northwestern Polytechnical University, Xi’an 710072, China; weitaofan@opt.ac.cn (W.F.);; 2 School of Science, Xi’an University of Posts & Telecommunications, Xi’an 710121, China

**Keywords:** self-trapped exciton, Ga_2_O_3_ film, dopant, photoluminescence, semiconductor

## Abstract

Doping divalent metal cations into Ga_2_O_3_ films plays a key role in adjusting the conductive behavior of the film. N-type high-resistivity *β*-Ga_2_O_3_:Mg films were prepared using electron beam evaporation and subsequent postannealing processing. Various characterization methods (X-ray diffraction, X-ray photoelectron spectroscopy, photoluminescence, etc.) revealed that the Mg content plays an important role in affecting the film quality. Specifically, when the Mg content in the film is 3.6%, the S2 film’s resistivity, carrier content, and carrier mobility are 59655.5 Ω·cm, 1.95 × 10^14^ cm^3^/C, and 0.53682 cm^2^/Vs. Also, the film exhibits a smoother surface, more refined grains, and higher self-trapped exciton emission efficiency. The Mg cation mainly substitutes the Ga^+^ cation at a tetrahedral site, acting as a trap for self-trapped holes.

## 1. Introduction

*β*-Ga_2_O_3_ is a promising semiconductor material with an ultra-wide band gap of about 4.9 eV, a critical electric field strength of about 8 MV·cm^−1^, excellent thermal and chemical stability, and an extremely high saturation mobility rate [1]. Its average transmittance rate in the UV–visible band is as high as 80%, which most traditional conductive oxides cannot reach in the UV region [2]. Due to the abovementioned characteristics, it has extraordinary application prospects in solar-blind UV photodetectors, sensors, and so on [2].

So far, the application of *β*-Ga_2_O_3_ is greatly limited by its conductivity, and especially *P*-type conductivity. Intrinsic *β*-Ga_2_O_3_ is usually an *N*-type semiconductor due to the contribution of the shallow donor levels of oxygen vacancies. *N*-type *β*-Ga_2_O_3_ is also relatively accessible by doping with tetravalent ions, such as Si [3] and Sn [4].

Theoretical studies [5] have reported that doping divalent ions, such as Mg^2+^ and Zn^2+^, to replace trivalent Ga^3+^ may obtain the *P*-type conduction of *β*-Ga_2_O_3_, although experimentally, it is relatively tough to achieve this. In fact, the role of Mg dopant in *β*-Ga_2_O_3_ is still in doubt. There are only several references that have reported hydrogen-induced high *P*-type conductivity in Ga_2_O_3_ [6], and weak *P*-type *β*-Ga_2_O_3_ thin films made through radio frequency magnetron sputtering followed by a postannealing treatment [7]. Also, there have been some studies that have not described the definite conduction behavior of *β*-Ga_2_O_3_. For example, Feng et al. [8] prepared Mg-doped *β*-Ga_2_O_3_ thin films on MgO (110) substrate using a metal–organic chemical vapor deposition technique and investigated its effect on the film’s properties using a post-deposition annealing method. Chu et al. [9] prepared Mg-doped Ga_2_O_3_ thin films with different Mg contents using a plasma-enhanced atomic layer deposition system. This dilemma may find one of its answers in the crystal structure of *β*-Ga_2_O_3_.

*β*-Ga_2_O_3_ belongs to the C*2*/*m* space group and has a base-centered monoclinic structure with four Ga_2_O_3_ formula units per 20 atom unit cells containing both tetrahedral GaO_4_ and octahedral GaO_6_ in equal quantities arranged in parallel chains [10,11]. In a unit cell, there are two different sites for Ga cations [Ga(I) and Ga(II)] and three different sites for O anions [O(1), O(2), and O(3)]. The Ga(I) (Ga^+^) and Ga(II) (Ga^3+^) are bonded to four and six neighboring O anions in the tetrahedral and octahedral arrangements, respectively. Most references [12,13,14,15,16,17,18] have claimed that a Mg ion replaces Ga^3+^ at the octahedral site, instead of Ga^+^ at the tetrahedral site. However, although Skachkov [5] agreed that Mg prefers the octahedral site, this might not completely exclude some Mg occurring in the tetrahedral site if the doping process has some nonequilibrium aspects. Therefore, it is quite necessary to further investigate the ionic occupation of Mg in *β*-Ga_2_O_3_.

According to the above references, other co-doping impurities [13,14,15,16,17,18], such as Fe^2+/3+^ or Zn^2+^, disturbed the doping effects of Mg cations. The purpose of this work is to prepare Mg-doped Ga_2_O_3_ thin films using electron beam evaporation. The electron beam evaporation method requires a very low film growth temperature and does not introduce any unintentional impurities, such as Fe, Si or Cr, into Ga_2_O_3_, like in the Czochralski method [13,14]. Thereafter, the effects of other impurities can be avoided and the effects of Mg on the electrical and optical properties of the films can be measured.

## 2. Materials and Methods

**Figure 1:**
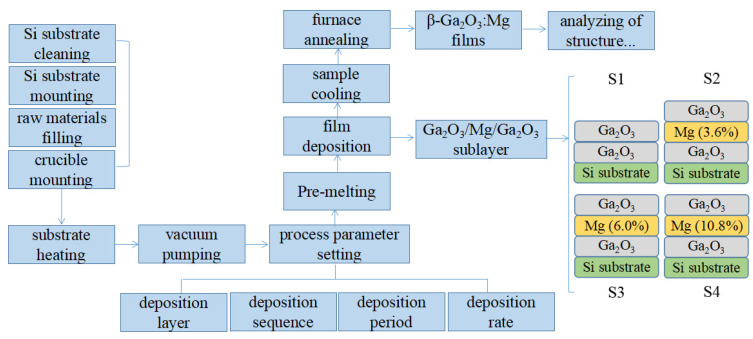
Flow chart of sample preparation processing.

Pre-cut 1 × 2 cm^2^ single crystalline silicon pieces were cleaned with acetone, deionized water, and alcohol solution in an ultrasonic cleaner for 15 min. Then, they were taken out of the solution, blown dry with an ear wash ball, and put on the sample holder of an electron beam evaporation system (model ZZS700, Xing-Nan Technology Co., LTD., Chengdu, China). The film deposition parameters (such as the substrate temperature, film deposition rate, sub-layer thickness, etc.) were set according to the deposition sequence of the Ga_2_O_3_-Mg-Ga_2_O_3_ triple sub-layers (like a “sandwich” structure) with a total film thickness of 150 nm. The doping content of Mg in the Ga_2_O_3_ film was nominally calculated according to Equation (1) [19], where *n* was the amount of Ga_2_O_3_ molecules in mol, *m* the mass of Mg, *M* the molar mass of Mg, *ρ* the density of Mg, *v* the volume, *A* the substrate area, and *H* the film thickness. The calculated molar ratio of Ga and Mg was approximately equal to the thickness ratio of Ga_2_O_3_ and Mg sub-layers. After determining the layer thickness, the Ga_2_O_3_ and Mg particles were, respectively, placed into two crucibles inside the vacuum chamber, and the Ga_2_O_3_ films with nominal Mg doping contents of 0, 3.6, 6.0, and 10.8% (referred to as S1, S2, S3, and S4) were obtained by adjusting the thickness of each sub-layer. After that, the samples were taken out of the vacuum chamber and annealed at 800 °C in a furnace in air for 10 h. The sample preparation process is summarized in Figure 1.

The phase compositions of the samples were determined using a Bruker D8 discover X-ray diffractometer (XRD) from Karlsruhe, Germany with an incidence angle of 1°, a scanning angle range of 25~70°, and a scanning rate of 10°/min. The surface morphology of the samples was evaluated using a ThermoFisher Quattro S scanning electron microscope (SEM) from Waltham, MA, USA. Thermo Scientific™ K-Alpha™ X-ray photoelectron spectroscopy (XPS) from Waltham, MA, United States was used to identify the presence of elements and their valence states in thin films. The photoluminescence (PL) properties of the films were measured at room temperature under an excitation light of 230 nm using a JASCO FP-8500 fluorescence spectrometer from Tokyo, Japan. The electrical properties of the samples were measured using a Hall effect system equipped with a PPMS-9 temperature-controlled field system, Keithley 2400 and Keithley2182 energy meters from San Diego, CA, United State, and the data acquisition was performed using Labview (win 2023 version) programming to obtain the carrier type, Hall coefficient, and carrier parameter.

(1) $$n=\frac{m}{M}=\frac{\rho v}{M}=\frac{\rho AH}{M}$$

## 3. Results and Discussion

**Figure 2:**
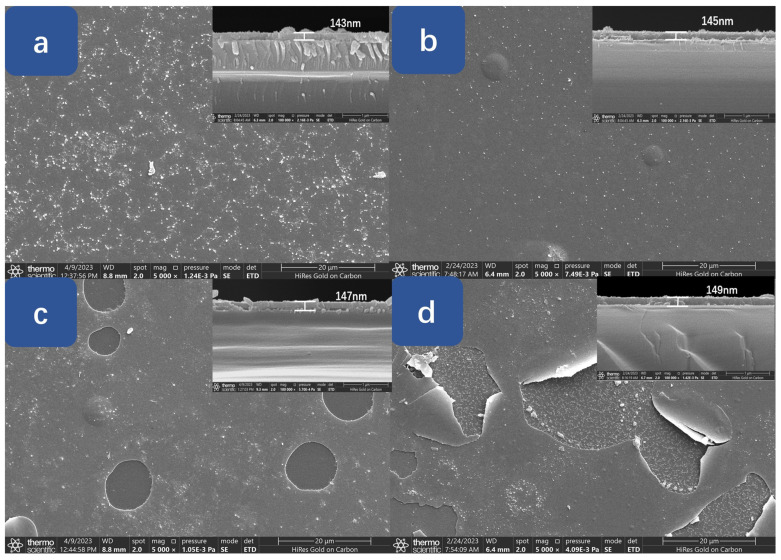
(**a**–**d**) Planar SEM images of S1–S4 films with their cross-sectional images (inset, upper-right corner).

Figure 2 shows the planar and cross-sectional SEM images of the S1–S4 films. As can be seen in the figure, the film morphologies are quite different: the S1 film surface in Figure 2a is very rough with fine particles randomly dotted on the surface. The morphology of the S2 film in Figure 2b is very dense with a flat surface and two rounded bumps are randomly distributed on it. In Figure 2c, large holes with a diameter range of 3~10 μm start to appear on the film surface. The Ga_2_O_3_ bottom layer surface is exposed inside the hole. The formation of these pores may be related to the thermal conductivity anisotropy of *β*-Ga_2_O_3_ films [1]. Except for these holes, the morphology of the S3 film is very similar with that of the S2 film and a rounded bump is observed on it too. In Figure 2d, some round Ga_2_O_3_ grains on the top layer of the “sandwich” structure peeled off or tended to peel off from the bottom layer. Plenty of small white dots (MgO grains) are observed inside the hole region and on the exposed Ga_2_O_3_ bottom layer surface. The inset images of Figure 2 are the cross-sectional images of the S1–S4 films. From these images, the thicknesses of the S1–S4 films are estimated to be 143.7, 145.7, 147.6, and 149.7 nm [20], respectively. Compared with our previous work of indium-doped Ga_2_O_3_ [1], the morphological evolution of Mg-doped Ga_2_O_3_ is very different: when the Mg content is only 3.6%, the film morphology is better than that of the undoped Ga_2_O_3_ film. However, more Mg addition will greatly deteriorate the film morphology. These results are due to the different mechanical behaviors between the Mg-doped and indium-doped Ga_2_O_3_ films.

**Figure 3:**
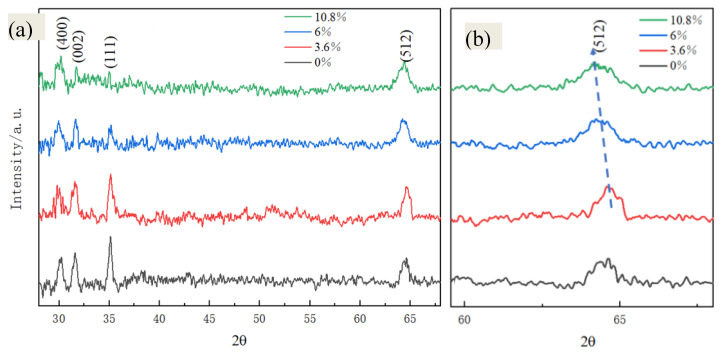
(**a**) The grazing incidence XRD patterns and (**b**) the amplified region containing (512) peaks of S1–S4 films.

The phase evolution of the films was thereafter achieved by XRD. Figure 3a shows the grazing incidence XRD patterns of the *β*-Ga_2_O_3_ films. The diffraction peaks are located at 30.181, 31.895, 35.268, and 64.541°, corresponding to the (400), (002), (111), and (512) grain orientations, respectively. The averaged grain size was calculated according to Scherrer’s equation:

(2) $$D=\frac{k\gamma }{Bcos\theta }$$

where *K* is Scherrer’s constant, *D* the average size of the grains, *B* is the full width at the half maximum value of the diffraction peak, *θ* is the angle of diffraction, and *γ* is the wavelength of the X-rays. The *B* values of the S1–S4 films are 0.498, 0.465, 0.513, and 0.567° for the (002) peak and 0.534, 0.435, 0.531, and 0.578° for the (111) peak. The averaged grain sizes of the S1–S4 films are 14.75, 16.92, 14.59, and 13.29 nm, respectively. The grain size tends to increase with the Mg content from 0 to 3.6%, and then reduces from 3.6 to 10.8%. That is, the crystalline quality is the best for the S2 film, and then becomes worse with greater Mg addition. In addition, Figure 3b shows the expanded (512) peaks of the four films, and the peak positions are located at 64.758, 64.752, 64.721, and 64.698° for S1–S4, respectively. As the addition of Mg increases from 3.6 to 10.8%, the (512) peak gradually shifts to the smaller diffraction angles, indicating that the lattice constants of the films are prone to becoming larger [7]. This phenomenon occurs probably because the theoretical ionic radius of Mg^2+^ (~0.72 Å) is larger than that of Ga^3+^ (~0.62 Å) or Ga^+^ (~0.47 Å) [21]; Mg cations probably enter the Ga_2_O_3_ lattice in the form of substitution ions for Ga cations. When more and more Mg cations replace Ga cations, the lattice constants expand, leading to the blue shift of the diffraction peaks [5,22]. Although the strain variation may also lead to the shift of the diffraction peaks [1], it may not be the reason for this in the Mg-doped Ga_2_O_3_ system. The film strain is calculated according to the following formula [1,23]:

(3) $$\varepsilon =\frac{d-d_{0}}{d_{0}}$$

where *ε* is the film strain, *d_0_* is the interplanar spacing of the stress-free film, and *d* is the interplanar spacing of the stressed film. The strain values of the *β*-Ga_2_O_3_ films are calculated and listed in Table 1. The table shows that the film strain values start to decrease from 1.185 × 10^−3^ to 0.358 × 10^−3^ with increasing Mg content, which is against the morphological variation trend of the films.

**Figure 4:**
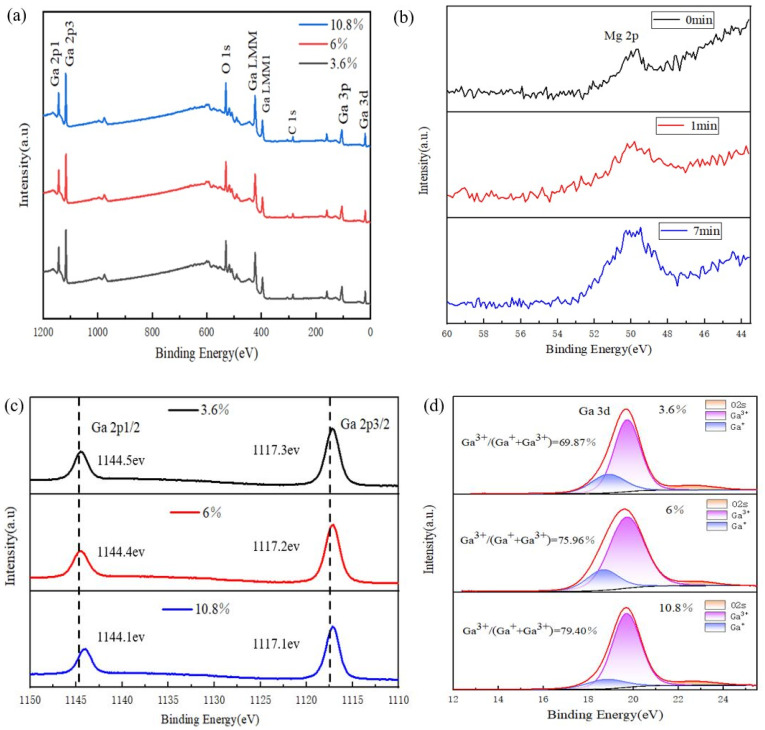

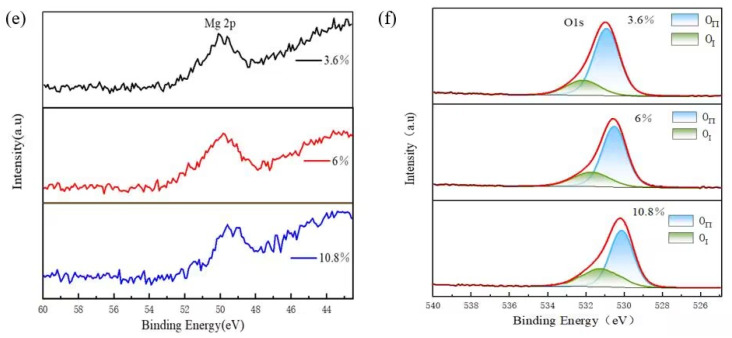
(**a**) XPS survey spectra of S2–S4 films; (**b**) XPS depth profiling of Mg element in S2 film; high resolution of (**c**) Ga 2p and (**e**) Mg 2p XPS spectra of S2–S4 films; and (**d**) Ga 3d and (**f**) O 1s XPS spectra of S2–S4 films with fitted subpeaks.

In order to obtain the elemental distribution on/inside the films with Mg content variation, XPS measurements were performed for the S2–S4 films. Figure 4a depicts the full XPS spectra of the different elements in the S2–S4 films. All the spectra were calibrated by referencing the C 1s peak at 284.8 eV, which is attributed to the hydrocarbon contamination on the film surface. The XPS peaks of O 1s, Ga 2p, Ga 3p, Ga 3d, Ga LMM, and Ga LMM1 are present in the figure. Figure 4b exhibits the variation in the Mg content in different depths of S2 film (with different Ar ion sputtering times of 0, 1, and 7 min, respectively). The Mg 2p peak intensity increasing with the sputtering times confirms the diffusion of Mg ions from the middle sub-layer of the sandwich structure up to the film surface, indicating that the metallic Mg sub-layer has been oxidized and Mg ions diffused throughout the film cross-section. Also, the intensity of the Mg 2p peaks increases with the film depth. This phenomenon suggests that the diffusion of Mg inside the film is not very uniform and the diffusion of Mg inside the film is a nonequilibrium process. The high-resolution XPS spectra of Ga 3d, Ga 2p, Mg 2p, and O 1s of the S2–S4 films are shown in Figure 4c–f. From Figure 4c, the positions of Ga 2p_1/2_ and Ga 2p_3/2_ are 1144.36 and 1117.43 eV for the S2 film, 1144.36 and 1117.43 eV for the S3 film, and 1144.10 and 1117.31 eV for the S4 film, respectively (the two dashed lines are the standard peak positions). The gaps between two peaks for the S2–S4 films are approximately 27 eV, which match well with the previously reported Ga 2p gap (26.9 eV) [8]. In addition, as the Mg content increases, both peak positions shift towards lower binding energy values, probably due to the redistribution of the charge around the Ga cations [8]. From Figure 4d, the low oxidation state Ga^+^ subpeak (the deep blue peak) implies the existence of oxygen vacancies (V_O_), which are caused by insufficient oxygen supply during the film formation. The area ratio of the Ga^3+^ subpeak (the purple peak) to the (Ga^+^+Ga^3+^) subpeaks increases with increasing Mg cation content [11], hinting that the Mg cation substitutes for Ga^+^ to some extent (see Table 2). Of course, the possibility of a Mg cation occupying the octahedral site cannot be excluded, as the diffusion of Mg from the middle sub-layer to the top sub-layer is a nonequilibrium process. The Mg 2p peaks in Figure 4e are located at 50.1 eV. Similar peak positions of Mg 2p have been reported in Mg-doped TiO_2_ [24] and Zn_1−x_Mg_x_O [25]. There is no metallic Mg peak observed, signifying that metallic Mg has been oxidized during film annealing. From Figure 4f, the green subpeaks belong to O_I_, which is due to the adsorption of C/O or OH^−1^ on the film surface [26,27]; the light blue subpeaks are attributed to O_II_, which are due to the lattice oxygen in the S2–S4 films [28]. The area ratio of the O_II_ subpeak (the purple peak) to the (O_I_+O_II_) subpeaks decreases with increasing Mg cation content, further disclosing that the oxidation states of the films are deteriorated (see Table 2) [20].

**Figure 5:**
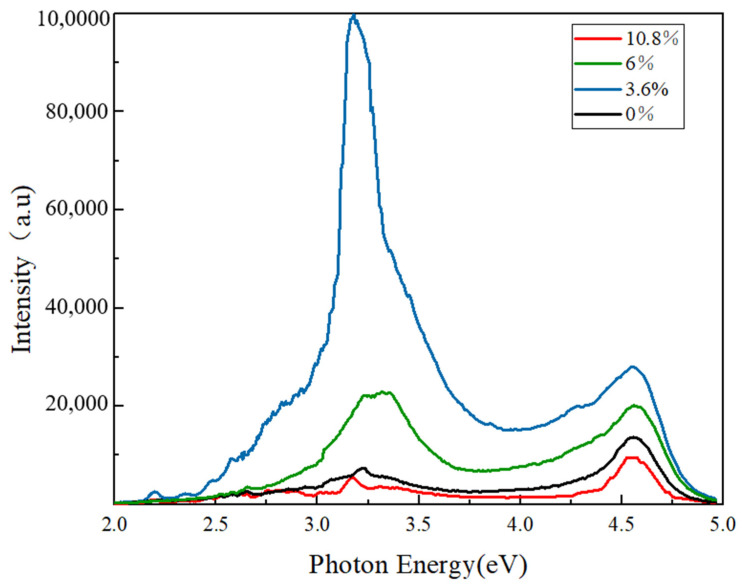
The PL spectra of Mg-doped *β*-Ga_2_O_3_ films annealed at 800 °C.

In order to evaluate the optical properties of the films, the PL spectra (see Figure 5) of the S1–S4 films were obtained in a wavelength range of 2 to 5 eV with an excitation wavelength of 230 nm (or a photon energy of 5.39 eV). Each of the PL curves has a narrow deep UV emission peak at 4.58 eV (or 270.7 nm) and a broad peak at 3.11~3.38 eV (or 398.7~366.9 nm). The intensity of the narrow peaks increases to the maximum for S2, and then gradually decreases from S2 to S4. The intensity variation of the broad peaks has a similar trend to that of the narrow ones, except their intensity variation is more extreme. The structure of the PL peaks with a broad peak with a large Stokes shift [29] and a narrow peak near to the conduction band–valence band emission are typical for self-trapped exciton (STE) emission [11,30,31].

An STE consists of a self-trapped hole (STH) and a bound electron [29]. In essence, an STH is a localized hole trapped by lattice distortions. In undoped *β*-Ga_2_O_3_ film, photogenerated holes can self-trap onto two different O sites (i.e., O_I_ and O_II_), referred to as STH_O1_ [12]. If some self-trapped holes are shared with two O, they are named STH_O2_. STH_O1_ or STH_O2_ can recombine with an electron at the conduction band minimum and a photon with the energy of 3.04 or 3.95 eV will be emitted. The experimental spectrum of STE emission falls between 3.1 and 3.6 eV, due to strong electron–phonon coupling.

When Mg is doped inside *β*-Ga_2_O_3_ film, it may replace Ga^+^ and Ga^3+^ cations, or act as an interstitial atom (Mg^0^). Previous XPS results have not identified a metallic Mg peak, thereby denying the existence of metallic Mg atoms in *β*-Ga_2_O_3_ film. If a Mg^2+^ cation replaces the Ga^3+^ cation as a polaronic acceptor (Mg_Ga2_) [12], the theoretical light emission spectra due to the Mg_Ga2_ acceptor turns into the visible light energy of 2.32 eV (for O_I_ site) or 2.53 eV (for O_II_ site). However, either peak does not appear in Figure 5. Combined with the XPS results, the most plausible site for the Mg replacement cation is the Ga^+^ site since there exists more Ga(I) vacancies than Ga(II) vacancies [11]. In fact, when Mg occupies the Mg_Ga1_ site, it may only lose one valence electron to maintain a charge balance. The leftover valence electron in the Mg cation plays the role of a trap for STH_O1_. Therefore, when a small amount of Mg (3.6%) is added into the film, the content of Mg_Ga1_ greatly increases, causing the PL peak at ~3.1 eV to increase to the maximum. When more Mg (6%) is added, the content of Mg_Ga1_ increases further; it becomes less possible for a photogenerated hole to self-trap onto one Mg_Ga1_ (the 3.1 eV peak intensity decreases), but more possible to share with two Mg_Ga1_ (i.e., STH_O2_). However, when excessive Mg (≥10.8%) is added, the crystalline quality becomes worse, and the photogenerated electrons may recombine with STHs non-radiatively, emitting several phonons [30]. This partially explains why there is only very weak STE emission for the S4 film in Figure 5. Another reason for the weak STE emission of the S4 film is ascribed to its Ga_2_O_3_ top layer peeling off the film surface.

The Hall effect is capable of determining the conduction type and the mobility of charge carriers in semiconductor materials. The film resistivity, carrier content, and carrier mobility of the S2 film are 59655.5 Ω·cm, 1.95 × 10^14^ cm^3^/C, and 0.53682 cm^2^/Vs. The S2 film exhibits N-type conduction. Even if some Mg cations occupy the Mg_Ga2_ site and form holes, these holes will lack the freedom to move, leading to an extremely high film resistivity and low carrier content.

## 4. Conclusions

In this work, a Ga_2_O_3_/Mg/Ga_2_O_3_ sandwich structure was deposited on Si substrates using electron beam evaporation, and then annealed in air to obtain N-type high-resistivity *β*-Ga_2_O_3_:Mg films. When the nominal Mg content was 3.6%, the film exhibited the best crystalline qualities, surface morphology, and STE emission spectra. Further adding Mg to the films deteriorated these properties. Mg occupying the Mg_Ga1_ site takes the main responsibility for these changes due to Mg_Ga1_ trapping holes and preventing the formation of free holes. In the future, theoretical investigations as well as experimental evidence, such as that from electron paramagnetic resonance spectroscopy, are needed in order to further clarify the occupation site of the Mg cation in Ga_2_O_3_ films.

## Figures and Tables

**Table 1:** The (512) peak’s positions and strains of S1–S4 films.

Sample No.	Peak Position (◦)	Strain ε (10^−3^)
S1 (0%)	64.758	1.185
S2 (3.6%)	64.752	1.103
S3 (6%)	64.721	0.675
S4 (10.8%)	64.698	0.358

**Table 2:** The area ratios of Ga^3+^/(Ga^3+^+Ga^+^) and O_II_/(O_I_+O_II_) for S2–S4 films.

Sample No.	Ga^3+^	Ga^+^	O_II_	O_I_	Ga^3+^/(Ga^3+^ + Ga^+^)	O_II_/(O_I_ + O_II_)
S2 (3.6%)	0.6987	0.3013	0.7745	0.2255	0.6987	0.7745
S3 (6%)	0.7596	0.2404	0.7285	0.2715	0.7596	0.7285
S4 (10.8%)	0.794	0.2006	0.6264	0.3736	0.7983	0.6264

## Data Availability

The original contributions presented in the study are included in the article, further inquiries can be directed to the corresponding author.
